# Traumeel S^® ^for pain relief following hallux valgus surgery: a randomized controlled trial

**DOI:** 10.1186/1472-6904-10-9

**Published:** 2010-04-12

**Authors:** Shepherd R Singer, Michal Amit-Kohn, Samuel Weiss, Jonathan Rosenblum, Guy Maoz, Noah Samuels, Esther Lukasiewicz, Laurence Freedman, Ora Paltiel, Menachem Itzchaki, Meir Niska, Menachem Oberbaum

**Affiliations:** 1 The Center for Integrative Complementary Medicine, Shaare Zedek Medical Center, Jerusalem, Israel; 2 Department of Orthopedics, Shaare Zedek Medical Center, Jerusalem, Israel; 3 Department of Orthopedics, Meir Medical Center, Kfar Saba, Israel; 4 Biostatistics Unit, Gertner Institute for Epidemiology and Health Policy Research, Chaim Sheba Medical Center, Tel Hashomer, Israel; 5 Faculty of Life Sciences, Bar-Ilan University, Ramat-Gan, Israel; 6 Department of Hematology, Hadassah University Hospital, and Hebrew University Hadassah School of Public Health, Jerusalem, Israel

## Abstract

**Background:**

In spite of recent advances in post-operative pain relief, pain following orthopedic surgery remains an ongoing challenge for clinicians. We examined whether a well known and frequently prescribed homeopathic preparation could mitigate post-operative pain.

**Method:**

We performed a randomized, double blind, placebo-controlled trial to evaluate the efficacy of the homeopathic preparation Traumeel S^® ^in minimizing post-operative pain and analgesic consumption following surgical correction of hallux valgus. Eighty consecutive patients were randomized to receive either Traumeel tablets or an indistinguishable placebo, and took primary and rescue oral analgesics as needed. Maximum numerical pain scores at rest and consumption of oral analgesics were recorded on day of surgery and for 13 days following surgery.

**Results:**

Traumeel was not found superior to placebo in minimizing pain or analgesic consumption over the 14 days of the trial, however a transient reduction in the daily maximum post-operative pain score favoring the Traumeel arm was observed on the day of surgery, a finding supported by a treatment-time interaction test (p = 0.04).

**Conclusions:**

Traumeel was not superior to placebo in minimizing pain or analgesic consumption over the 14 days of the trial. A transient reduction in the daily maximum post-operative pain score on the day of surgery is of questionable clinical importance.

**Trial Registration:**

This study was registered at ClinicalTrials.gov. # NCT00279513

## Background

The management of postoperative pain following ambulatory orthopedic surgery is an issue of ongoing concern for patients and for physicians performing these procedures. Numerous studies have found pain control to be inadequate [1-6]. Pain is the most frequent cause of delayed discharge and unanticipated readmission following ambulatory surgery [7,8], as well as for contacting the family physician after discharge [9]. Nearly a third of patients have moderate to severe pain 24 hours after ambulatory surgery [10], while eleven percent experience severe pain [11]. Both moderate and severe pain can last for up to a week after surgery [9]. Treatment for post-operative pain typically includes anti-inflammatory medications and opiates, both of which are associated with adverse effects, limiting patient compliance and effectiveness.

Traumeel S^® ^is an over-the-counter homeopathic preparation composed of extracts from a combination of plants and minerals that have been highly-diluted, though not beyond Avogadro's number (see Table 1). It has been widely sold in German, Switzerland and Austria for over 50 years, and is one of the most popular alternative medications in these countries, selling approximately four million doses a year [manufacturer information]. Earlier studies have suggested that Traumeel may be effective in trauma [12-15], acute tendinopathy [16] and in the spinal syndrome [17], though these studies were either non-randomized or used poorly chosen controls. A small RCT found Traumeel to be effective in post-chemotherapy stomatitis [18]. While the mechanism of action of this preparation remains unknown, recent research has shown that Traumeel reduces secretion of pro-inflammatory cytokines from various human immune cells in vitro, both at rest and when activated by PHA-, PMA-, or TNF-α. Interleukin-1β secretion was reduced by 70%, TNF-α by 65% and 54% (resting and activated), and IL-8 by 50% (P < 0.01 for all comparisons) [19].

**Table 1:** Composition of the homeopathic-complex Traumeel S^®^.

Component	Homeopathic dilution	Quantity per tablet
Arnica Montana	D2	150 μg

Calendula officinalis	D2	150 μg

Atropa belladonna	D4	7.5 μg

Aconitum napellus	D3	30 μg

Bellis perennis	D2	60 μg

Hypericum perforatum	D2	30 μg

Echinacea angustifolia	D2	60 μg

Echinacea purpurea	D2	60 μg

Hepar sulfuris	D8	300 pg

Symphytum officinale	D8	240 pg

Matricaria Chamomilla	D3	24 μg

Achillea millefolium	D3	15 μg

Mercurius solubilis Hahnemanni	D8	300 pg

μg = microgram (1/1,000,000 g), pg = picogram (1/1,000,000,000 g)

Unpublished data from a manufacturer safety survey indicated adverse events in 0.0035% of 3.5 million cases [personal communication]. These appeared almost entirely in cases where Traumeel was injected, with the most common of these events being local irritation at the site of injection that resolved spontaneously after discontinuation of treatment. No drug interactions are known with this preparation [manufacturer information].

In 2007 we published the results of an open pilot study to evaluate the efficacy of Traumeel in mitigating post-operative pain following ambulatory hallus valgus surgery [20]. In this study, treatment allocation was by week of surgery rather than by strict randomization. This study found significantly lower post-operative pain scores in patients treated with supplementary Traumeel as compared with standard care alone. To the best of our knowledge, no randomized controlled trial has yet assessed the effectiveness of Traumeel for the relief of acute pain in patients following ambulatory surgery. We therefore performed a randomized, double-blind, placebo-controlled clinical trial to evaluate the efficacy of Traumeel in controlling post-operative pain after ambulatory hallus valgus correction.

## Methods

### Study design

The study was designed, conducted, and reported according to the Consolidated Standards of Reporting Trials (CONSORT) guidelines [21], and approved by the ethics committees of the Shaare Zedek Medical Center (SZMC) and the Meir Medical Centers (MMC). All patients scheduled for ambulatory hallus valgus surgery at the Shaare Zedek Medical Center department of orthopedics between 12 September 2006 and 19 November 2007, and at Meir Medical Center between 1 August 2007 and 19 November 2007 were screened for inclusion in the trial. The exclusion criteria were as follows: age under 18 years, bilateral surgery, previous hallus valgus surgery on the ipsilateral foot, diseases possibly effecting wound healing or pain sensation (uncontrolled diabetes mellitus, Berger's disease, deep vein thrombosis, peripheral vascular disease), sensitivity to any of the study medications and technical or cognitive inability to comply with the study protocol. Eighty consecutive patients who fulfilled all inclusion and no exclusion criteria and who signed informed consent were enrolled in the trial.

Study design was randomized and double-blind. Randomization blocks of four subjects were created, and then selected using random number sequence. The HEEL Company (Baden-Baden, Germany) prepared and supplied the study medication or an indistinguishable placebo in 100 consecutively numbered, sealed boxes, according to the randomization list. Sealed copies of the randomization list were held by the manufacturer and a hospital physician not otherwise involved in the trial. These were to be opened only in the event of a medical emergency necessitating knowledge of the treatment allocation of a given patient. Otherwise, patient and all study personnel remained unaware of treatment allocation until the trail was completed and the database sealed, at which point the allocation envelopes were to be opened and the randomization code unveiled. Each eligible patient was assigned the lowest numbered box available. Surgery was performed as orthopedically indicated, with all patients anesthetized using an ankle block of 20 cc Lidocaine 1% and Marcaine 0.5%. Upon completion of surgery, patients were instructed to take two tablets of the study medication five times daily for the first three days, and three times daily afterwards. The composition of Traumeel tablets is displayed in Table 1. Patients also received tablets of paracetamol 325 mg with codeine 15 mg (Cod-Acamol Forte, Teva Pharmaceutical Industries, Israel) as primary analgesic, to be taken in a two-tablet dose up to six times daily, as needed. Patients additionally received a prescription for Tramadol tablets 100 mg (Tramadex, Dexcel Pharma, Israel) as rescue analgesic to be taken as needed, up to a maximum of four tablets a day. The choice of a primary analgesic including a mild opiate was based upon the necessity for standardized primary analgesia while offering patients adequate pain relief over the course of the trial. Previous experience had indicated that non-opiate analgesics were insufficient for this purpose. Patients were instructed to record maximal daily pain scores at rest using a self-administered, horizontal 11-point numerical rating score (NRS-11), graded from 0 (no pain) to 10 (worst imaginable pain). Patients were further requested to record in the patient diary the number of primary and rescue analgesic tablets taken, and the maximum level of pain at rest they had experienced during that day.

Patients were contacted by telephone every evening by a study nurse, who recorded the reported NSR and analgesic consumption values in the case report form. The nurse also encouraged study compliance. Patients were instructed to withhold study medication if the NRS score was three or less and no other analgesics had been consumed for two consecutive days. Patients were examined by a surgeon on days 6 and 13 days after surgery, and any adverse events, related or unrelated to treatment, were registered. On post-operative day 13, all study materials were collected from the patient.

### Efficacy measures

The primary outcome measure was maximum daily pain at rest, as measured by a horizontal, 11-point numerical rating scale (NRS-11) filled out on the day of surgery (day 0) and for 13 post-operative days. Secondary outcomes were total consumption of primary analgesics and number of days requiring rescue analgesics. Adverse events were monitored throughout the study.

### Statistical analysis

Statistical analysis was performed following the intention-to-treat principle. To compare the post-operative pain between the study groups, the area-under-the-curve (AUC) of the NRS pain scores recorded daily over the 2 weeks post-operative was used. We chose the AUC for both clinical and statistical reasons. Clinically, it summarizes the strength of pain and the number of days the patient experienced pain. Statistically, it is a summary measure which allows one to deal with repeated measurements with no need to adjust for the type 1 error rate. For each patient, an AUC was obtained by graphing the NRS pain scores recorded every day during follow-up, linking time-adjacent points by a straight line and calculating the area under the resulting polygon. When all 14 pain scores are available, this is equivalent to the sum of the 14 scores. Linear interpolation was performed to estimate the NRS score on those days that were missing. The mean AUCs were compared between study groups using the Mann-Whitney test. The repeated measures mixed model was used to test for an interaction between time and treatment allocation. Mann-Whitney tests were used to compare the total amount of primary analgesics consumed and the total number of days taking rescue analgesics. Post-hoc subgroup analysis was performed to assess the influence of type of surgery on post-operative pain scores. Surgical procedures were characterized as 'Chevron only', 'Additional osteotomy', or 'Triple or proximal osteotomy', and Mann-Whitney tests were performed in each subgroup to compare the area-under-the-curve of the NRS pain scores between treatment arms. Frequency of the main adverse events was compared using Fisher's exact test. Sample size was calculated using a conservative interpretation of the data obtained in our previous pilot study [20], a type I error of 0.05, a power of 90% and a 15% loss to follow-up.

## Results

**Figure 1:**
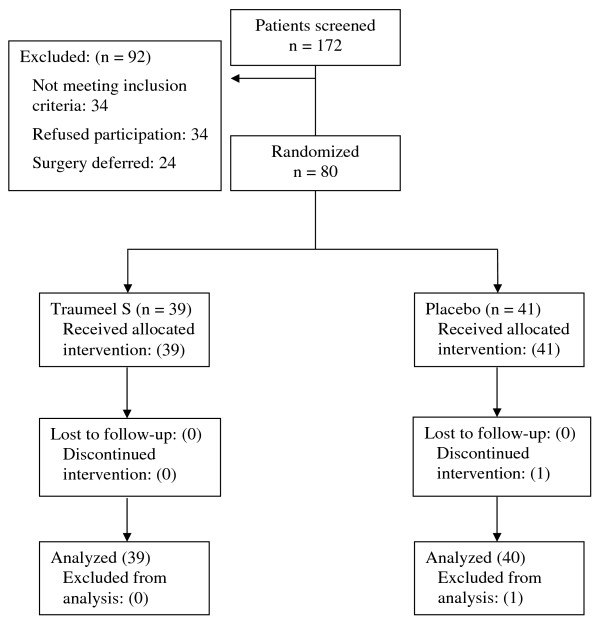
**Flow chart of patients through study**. 172 patients were screened for participation in the trial. Of 80 patients randomized, one discontinued treatment and was excluded from analysis.

Patient flow throughout the study is presented in Figure 1. Of the 80 patients who participated in the trial, 79 were evaluable for intention-to-treat analysis. The remaining patient withdrew consent on the third day of the trial because of nausea, vomiting and abdominal pain. He did not provide sufficient data points to be included in the final analysis. Patient groups were balanced with respect to all baseline parameters except for laterality of surgery (Table 2).

**Table 2:** Baseline demographic and clinical variables of the Traumeel S^® ^and placebo treatment groups.

	Traumeel S^®^ (n = 39)	Placebo (n = 40)
Clinical Center:		
SZMC*	33	34
MMC**	6	6
Age (y)		
Mean (SD)	48.1 (16.7)	45.2 (18.7)
< 50	17	19
≥ 50	22	21
Sex (%)		
M	8 (20.5)	8 (20.0)
F	31 (79.5)	32 (80.0)
Ethnicity/Origin (%)		
Europe/America	12 (30.8)	9 (22.5)
Asia/Africa	21 (53.9)	25 (62.5)
Israeli Arab	2 (5.1)	0
Mixed	2 (5.1)	2 (5.0)
Other	2 (5.1)	4 (10.0)
BMI		
Mean (SD)	24.7 (4.1)^†^	24.3 (4.0)^†^
< 24	20	23
≥ 24	18	16
Pain before surgery		
Mean (SD)	5.4 (2.8)	5.7 (2.6)^†^
< 6	19	18
≥ 6	20	21

Laterality (%)		
Right	21 (53.9)	14 (35.9)^†^
Left	16 (41.0)	24 (61.5)
Bilateral^#^	2 (5.1)	1 (2.6)

Type of surgery (%)		
Chevron - only	9^† ^(23.0)	10^†† ^(26.3)
Additional	17 (43.5)	18 (47.3)
osteotomy		
Triple or	13 (33.3)	10 (26.3)
proximal		
osteotomy		

* SZMC -- Shaare Zedek Medical Center

**MMC -- Meir Medical Center

^#^3 patients had a bilateral hallux valgus but underwent surgery on only one foot.

^† ^- includes 1 patient with missing data.

^††-^includes 2 patients with missing data.

**Figure 2:**
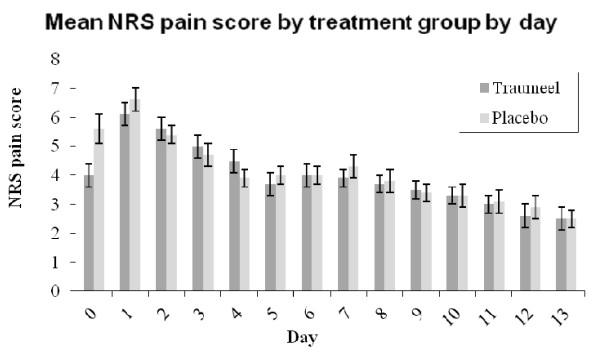
**Mean pain score over the 14 days of the trial**. Mean NRS pain scores over 14 days for 79 patients receiving Traumeel S^® ^or Placebo. The error bars indicate the mean plus or minus 1 standard error.

Overall, the mean area-under-the-curve pain scores during the 14 days of the trial were not significantly different between the groups (55.4 ± 25.5 in Traumeel group vs. 57.4 ± 25.7 for placebo, p = 0.89). However, the mean pain score on the day of surgery appeared lower in the Traumeel group than placebo (4.0 vs. 5.6, Figure 2). This difference is supported by the test for interaction between time and treatment group (p = 0.04).

When stratified for type of surgery, in the Chevron-only strata the mean pain score appeared lower in the Traumeel group than in the placebo group, however this difference was not found statistically significant (Traumeel - 46.1, Placebo - 61.8, p = 0.35). In the 'Additional osteotomy' and ' Triple or proximal osteotomy' groups, differences in mean pain between the verum and placebo groups were negligible (Table 3).

**Table 3:** Pain score stratified for type of surgery.

	Chevron-only		Additional osteotomy		Triple or proximal osteotomy	
**AUC**	**Traumeel (n = 9)**	**Placebo (n = 10)**	**Traumeel (n = 17)**	**Placebo (n = 18)**	**Traumeel (n = 13)**	**Placebo (n = 10)**

Mean	46.1	61.8	63.1	59.5	51.6	49.2

SD	23.4	34.1	25.6	55.5	49.0	49.0

p-value	0.35		0.69		0.88	

AUC -- Area Under the Curve

There was little difference between the two arms in the total number of tablets of primary analgesics consumed (15.6 ± 12.2 for Traumeel vs. 16.0 ± 11.9 for placebo, p = 0.74) and in the number of days on which rescue analgesics were required (mean ± SD: 1.0 ± 3.1 for Traumeel and 1.5 ± 2.0 for Placebo, p = 0.99).

Four patients in the placebo group (10%) and two in the Traumeel group (5.1%) developed wound infection. Six patients in the Traumeel group (15%) and four patients in the placebo group (10%) developed nausea and/or vomiting, though these differences were not statistically significant (p = 0.51 and p = 0.68 respectively). In the Traumeel group, one 82-year-old patient with type II diabetes developed cellulitis following wound infection. He was hospitalized for intravenous antibiotic treatment, but did not require further surgery. This was deemed a serious adverse event unrelated to the study medication.

## Discussion

Traumeel was not superior to placebo in minimizing pain or analgesic consumption over the 14 days of the trial, however a transient reduction in the daily maximum post-operative pain score was observed on the day of surgery. Our statistical design did not allow for a significance test specific to that day, however a test for interaction was significant. Stratification by type of surgery did not alter these results. Because post-operative pain is maximal in the period immediately following surgery, this finding may be of clinical importance. Wound infection was more prevalent in the placebo group, but the difference was not statistically significant. Other adverse effects were too rare to analyze statistically.

Our findings do not support those of a pilot study published in 2007 that suggested a 30% improvement in pain over 14 post-operative days, and a non-significant trend towards lower analgesic consumption. In that non-blinded study, 30 subjects were allocated by week of surgery to intraoperative injection of Traumeel, injection plus oral Traumeel, or no treatment. Both treatment arms were found superior to no treatment [20]. Differences in study methodology could explain the differences in outcome. We believe that future research should focus on the early post-operative period.

Homeopathy is frequently attacked for its use of solutions diluted beyond Avogadro's number, and hence physical-chemical implausibility. That criticism was circumvented in this trial by employing a preparation of solutions that were dilute, but well within the material range.

Relief of post-operative pain remains an ongoing challenge. While other treatment modalities have been examined, each has its own limitations. Valdecoxib and rofecoxib have both been found effective for relief of pain following hallux valgus surgery [22,23] but were removed from the market amid concerns of excessive risk of heart attack and stroke. Betamethasone has been found to reduce post-operative pain and nausea [24], but concerns remain regarding the effect of corticosteroids on post-operative healing [25]. Parenteral routes for delivery of opiates are available, but they entail greater cost, complexity, and possible need for hospitalization [26].

This trial has several limitations. By choosing a cumulative 14-day measure for our primary outcome, we may have inadvertently diluted any effect that may have been present in the first days after surgery - those with the greatest pain. Homeopathic purists may find fault in the administration of a standardized combination homeopathic formula to all patients, based upon clinical diagnosis - as opposed to the individualized manner dictated by standard homeopathic practice. We were aware of this limitation at the outset; however, performing an individualized RCT would be far more complex, time-consuming, and fraught with methodological pitfalls. The mode of administration of the remedy may have played a role as well. In contrast to our pilot study, in the current study, for the sake of simplicity we chose to use only oral administration of the study medication, under the assumption that its effect would be similar to that of injection followed by oral therapy. In retrospect, that assumption may have been mistaken.

## Conclusions

The homeopathic complex Traumeel S^® ^was not found superior to placebo in mitigating post-operative pain or analgesic consumption over the 14 days of the trial. A transient reduction in the daily maximum post-operative pain score, observed on the day of surgery, is of questionable importance. We recommend repetition of this trial, but using injected Traumeel and focusing on the early post-operative period.

## Abbreviations

CONSORT: Consolidated Standards of Reporting Trials; MMC: Meir Medical Center, Kefar Saba, Israel; NRS: Numerical Rating Scale; SZMC: Shaare Zedek Medical Center, Jerusalem, Israel.

## Competing interests

The HEEL Company (Baden-Baden, Germany) has funded previous research at the Shaare Zedek Center for Integrative Complementary Medicine, and provided funding for the performance and monitoring of this project, supplied the study medication and placebo, and prepared the randomization list. However, it had by contractual agreement no control over the flow of the study, the data analysis, or the decision when and where to publish the study findings. The authors have full control over the primary data, and agree to allow the journal to review the data if requested.

## Authors' contributions

MO conceived the trial, and was central to its design, performance, and reporting.

SRS was PI, participated in drafting the protocol, oversaw performance of the trial and participated in drafting the manuscript. NS and OP participated in study design and drafting of protocol and manuscript. MAK, SW, JR, GM, MI and MN screened patients and performed surgery and follow-ups. EL and LF participated in design of the trial, data management and analysis. All authors have read and approved the final manuscript.

SRS and MAK are joint first authors.

## Pre-publication history

The pre-publication history for this paper can be accessed here:

http://www.biomedcentral.com/1472-6904/10/9/prepub
